# *Indopolystoma* n. gen. (Monogenea, Polystomatidae) with the description of three new species and reassignment of eight known *Polystoma* species from Asian frogs (Anura, Rhacophoridae)

**DOI:** 10.1051/parasite/2019067

**Published:** 2019-11-20

**Authors:** Amira Chaabane, Olivier Verneau, Louis Du Preez

**Affiliations:** 1 Unit for Environmental Sciences and Management, North-West University, Potchefstroom Campus Private Bag X6001 2520 Potchefstroom South Africa; 2 University of Perpignan Via Domitia, Centre de Formation et de Recherche sur les Environnements Méditerranéens, UMR 5110 66860 Perpignan France; 3 CNRS, Centre de Formation et de Recherche sur les Environnements Méditerranéens, UMR 5110 66860 Perpignan France; 4 South African Institute for Aquatic Biodiversity Private Bag 1015 6140 Grahamstown South Africa

**Keywords:** *Indopolystoma*, *Polystoma*, Asia, Neobatrachia, Rhacophoridae

## Abstract

The polystomes (Monogenea, Polystomatidae) radiated across semi-aquatic tetrapods including all three amphibian orders, freshwater turtles and the hippopotamus. Prior to this study, phylogenetic analyses revealed that the most diverse and widespread genus, *Polystoma*, was not monophyletic; a lineage comprising four undescribed species from the bladder of *Zhangixalus* spp. (Rhacophoridae) in Asia occupied a deep phylogenetic position. Regarding vicariance biogeography and molecular dating, the origin of this lineage is correlated with the breakup of Gondwanaland in the Mesozoic period. Based on a Bayesian analysis of four concatenated genes (18S, 28S, COI and 12S) and morphological evidence, one new genus, *Indopolystoma* n. gen., and three new species, sampled in Japan and China, are described here: *Indopolystoma viridi* n. sp. from *Z. viridis* of Japan, *Indopolystoma elongatum* n. sp. from *Z. arboreus* of Japan, and *Indopolystoma parvum* n. sp. from *Z. omeimontis* of China. *Indopolystoma* is unique amongst polystome genera infecting anurans by possessing a small haptor relative to the body size, posteriormost marginal hooklet C1 much bigger than hooklets C2–C8 with conspicuous broad blade and guard and a pair of hamuli lacking a deep notch. Eight species of Asian *Polystoma*, all from rhacophorids, are transferred as *Indopolystoma carvirostris* (Fan, Li & He, 2008) n. comb., *I. hakgalense* (Crusz & Ching, 1975) n. comb., *I. indicum* (Diengdoh & Tandon, 1991) n. comb., *I. leucomystax* (Zhang & Long, 1987) n. comb., *I. mutus* (Meng, Song & Ding, 2010) n. comb., *I. pingbianensis* (Fan, Wang & Li, 2004) n. comb., *I. rhacophori* (Yamaguti, 1936) n. comb., and *I. zuoi* (Shen, Wang & Fan, 2013) n. comb.

## Abstract (fr)

Les polystomes (Monogenea, Polystomatidae) se sont diversifiés sur des tétrapodes semi-aquatiques, notamment les trois ordres d’amphibiens, les tortues d’eau douce et l’hippopotame. Avant cette étude, des analyses phylogénétiques avaient révélé que le genre le plus diversifié et le plus répandu, *Polystoma*, n’était pas monophylétique ; une lignée comprenant quatre espèces non décrites de la vessie de *Zhangixalus* spp. (Rhacophoridae) en Asie occupait une position phylogénétique profonde. En ce qui concerne la biogéographie de vicariance et la datation moléculaire, l’origine de cette lignée est corrélée à l’éclatement du Gondwana au Mésozoïque. D’après une analyse bayésienne de quatre gènes concaténés (18S, 28S, COI et 12S) et des preuves morphologiques, un nouveau genre, *Indopolystoma* n. gen. et trois nouvelles espèces échantillonnées au Japon et en Chine sont décrites ici : *Indopolystoma viridi* n. sp. de *Z. viridis* du Japon, *Indopolystoma elongatum* n. sp. de *Z. arboreus* du Japon et *Indopolystoma parvum* n. sp. de *Z. omeimontis* de Chine. *Indopolystoma* est unique parmi les genres de polystomes infectant les anoures. Il possède un hapteur petit par rapport à la taille du corps, un crocheton le plus postérieur C1 beaucoup plus gros que les crochetons C2 à C8, avec lame et garde bien visibles, ainsi qu’une paire d’hamuli dépourvus d’encoche profonde. Huit espèces de polystomes asiatiques, toutes issues de Rhacophoridae, sont transférées comme *Indopolystoma carvirostris* (Fan, Li & He, 2008) n. comb., *I. hakgalense* (Crusz et Ching, 1975) n. comb., *I. indicum* (Diengdoh & Tandon, 1991) n. comb., *I. leucomystax* (Zhang & Long, 1987) n. comb., *I. mutus* (Meng, Song & Ding, 2010) n. comb., *I. pingbianensis* (Fan, Wang et Li, 2004) n. comb., *I. rhacophori* (Yamaguti, 1936) n. comb. et *I. zuoi* (Shen, Wang et Fan, 2013) n. comb.

## Introduction

In contrast to the digeneans that can be found in all groups of vertebrates, monogeneans are mostly parasites of marine and freshwater fishes [52]. With the exception of a few monogeneans that were assigned to the Gyrodactylidae Cobbold, 1864, Iagotrematidae Mañé-Garzón & Gil 1962, and Lagarocotylidae Kritsky, Hoberg & Aubry, 1993, only a single family, the Polystomatidae Gamble, 1896, radiated across semi-aquatic tetrapods including all three amphibian orders (anurans, salamanders and caecilians), freshwater turtles and the common hippopotamus. The Polystomatidae, in modern classification, belong to the order Polyopisthocotylea Odhner, 1912. Nowadays, polystomatids are globally in excess of 180 described species in 26 genera, most of which are endo-parasitic in the bladder of amphibian adults (18 genera) and in the pharyngeal cavity, bladder or conjunctival sacs of freshwater turtles (five genera). Whereas fish usually harbor a high diversity of monogeneans on their gills [28, 41], no more than two species of polystomes have thus far been recorded per species of anuran host [7, 15]. Finally, a high degree of host-specificity was assumed for polystomatids of especially anuran hosts (see [49] for a review on the diversity of polystomatids).

The Polystomatidae thus provided the opportunity to trace host-parasite co-evolution over an exceptionally long period of time, namely from the ecological transition from marine to terrestrial life at about 425 million years ago (Mya) [50]. Whereas flatworm groups often display diverse body plans, monogeneans and in particular polystomatids show limited interspecies variation [45]. Although hardly any information is known about ancestral forms, the molecular phylogenies published in Bentz et al. [4, 5], Verneau et al. [50, 51], Badets et al. [2] and Héritier et al. [22] gave an invaluable timescale to date evolutionary events and to infer origins of major monophyletic groups within the family. The extant anuran polystomatids in Asia are less than 20 species that belong to five genera, *Diplorchis* Ozaki, 1931, *Eupolystoma* Kaw, 1950, *Neoriojatrema* Imkongwapang & Tandon, 2010, *Polystoma* Zeder, 1800 and *Sundapolystoma* Lim & Du Preez, 2001. Of these, the ubiquitous *Polystoma*, which is the most speciose-polystome genus known from anurans of the suborder Neobatrachia Reig, 1958, encompasses 14 parasite species (six from China, four from Japan and one each from Sri Lanka, India, Iran and Turkey). Earlier studies based on the phylogeny and historical biogeography of polystomes infecting species of the Neobatrachia [2] revealed that *Polystoma* was not a monophyletic taxon and that the deep-branched lineage including *Polystoma* species sampled from rhacophorids of India, Japan and China was strongly correlated with the breakup of Gondwanaland in the Mesozoic period. Badets et al. [2] suggested from cophylogenetic and vicariance analyses supplemented by molecular dating that this lineage probably arose on the Indian subcontinent about 177 Ma when western and eastern Gondwanan components were fully separated, and later colonized southeast Asia following host dispersal after India collided with Asia close to 86 Mya.

India is one of the largest landmass countries in Asia and also well-recognized as a rich biogeographic area in terms of species diversity and endemic species, with its boundaries falling in Himalaya, Western Ghats, Indo-Burma and Sundaland biodiversity “hot spots” [34, 37]. For instance, India has a striking anuran diversity with 395 known species [21] of which 286 (73%) are endemic [1]. As shown by many authors, the geological history of India played a crucial role in shaping the current diversity, endemicity, and distribution patterns of amphibian lineages. Before joining Laurasia, India was part of Gondwanaland and gradually became detached from other landmasses during its northward journey across the Tethys Sea [29]. It broke off from Africa about 130 Mya [29] and subsequently from Madagascar about 88 Mya [42]. Its collision with southern Asia occurred during the Paleocene or Early Eocene at 66–56 Mya [3] and gave rise to biotic exchange [6].

According to plate tectonics, rifting and drifting of continents following the breakup of Gondwana provided ample time for animal differentiation. Therefore, the long period of isolation of the *Polystoma* lineage in the Indian subcontinent [2] should have been sufficient to restrict specific morphological marks for this higher taxon. In the present study, we focused on several specimens of the three undescribed Asian *Polystoma* species reported in Verneau et al. [51], Badets et al. [2] and Héritier et al. [22] to provide formal descriptions of this new taxon and species. Based on genetic and morphological characters, we bring some evidence that this lineage, which includes polystomes of Asian rhacophorids, is a new genus within the Polystomatidae, and we also reassign eight polystomes previously described as *Polystoma* to this genus.

## Materials and methods

### Polystome sampling and morphology

Polystomes were recovered from the bladder of three Asian rhacophorids belonging to *Zhangixalus* Jiang et al. 2019 [21, 27], namely *Z. viridis* (Hallowell) and *Z. arboreus* (Okada & Kawano) that were both collected in Japan by Hideo Hasegawa on 8 February 1986, and 27 June 2003, respectively, and *Z. omeimontis* (Stejneger) that was collected in China by Annemarie Ohler on 11 May 2004. A single parasite specimen from each host species was fixed in alcohol for molecular analyses and processed in Badets et al. [2] and Héritier et al. [22]. Whereas some of the material collected in Japan was stained and mounted in Canada balsam, all specimens collected in China were preserved in alcohol. We therefore stained all of them but one with acetocarmine and mounted them permanently in Canada balsam. Specimens were examined using a Nikon NiE compound microscope (Nikon, Netherlands) fitted with a Nikon DS-Ri1 digital camera and drawn using Adobe Illustrator software. Measurements were taken, in micrometers, using a Nikon NIS elements D software program and expressed as the mean, followed by the range in parentheses.

### Sequence collection

In order to establish the phylogenetic relationships of the polystomes assumed to belong to a new genus, namely polystomes recovered from *Zhangixalus* frogs, we selected the four Asian *Polystoma* species reported in Badets et al. [2] and Héritier et al. [22] as well as one to three species of the main genera infecting neobatrachian frogs, namely *Diplorchis ranae* Ozaki, 1931, *Eupolystoma alluaudi* (de Beauchamp, 1913), *Kankana manampoka* Raharivololoniaina et al. 2011, *Madapolystoma* sp., *Parapolystoma bulliense* (Johnston, 1912), *Polystoma cuvieri* Vaucher, 1990, *P. gallieni* Price, 1939 and *P. naevius* Caballero & Cerecero, 1941, for which sequences were available in GenBank. Two other species were also selected for outgroup comparisons according to Héritier et al. [22], namely *Pseudodiplorchis americanus* (Rodgers & Kuntz, 1940) and *Pseudopolystoma dendriticum* (Ozaki, 1948). All these species with their respective accession numbers for two nuclear (18S and 28S) and two mitochondrial (12S and COI) genes are reported in Table 1. Prior to running phylogenetic analyses, we noticed that COI sequences reported in Héritier et al. [22] for polystomes infecting *Rhacophorus maximus* Günther (known today as *Zhangixalus smaragdinus* (Blyth)) (JF699303) and *Z. viridis* (KR856171) were almost identical, differing by only two substitutions, while pairwise comparisons of 12S, 18S and 28S sequences showed higher molecular divergences. This suggested inversion of DNA samples during the amplification process. Using primers L-CO1p and H-Cox1R and following the PCR procedure described in Héritier et al. [22], we therefore re-amplified the COI fragment from both polystome DNA samples recovered by these authors and selected the new sequences for phylogenetic and genetic analyses.

**Table 1:** Species of polystomes investigated with their 18S, 28S, 12S and COI GenBank Accession numbers. *Indopolystoma* spp. were considered earlier as *Polystoma* spp. in Verneau et al. [51], Badets et al. [2] and Héritier et al. [22].

Polystome species	Host species	Family	Country	18S	28S	12S	COI
*Diplorchis ranae*	*Glandirana rugosa*	Ranidae	Japan	AM157184	AM157198	KR856070	JF699304
*Eupolystoma alluaudi*	*Bufo* sp.	Bufonidae	Togo	AM051066	AM157199	KR856072	FR667558
*Kankana manampoka*	*Platypelis pollicaris*	Microhylidae	Madagascar	HM854292	HM854293	KR856074	JF699307
*Madapolystoma* sp.	*Blommersia wittei*	Mantellidae	Madagascar	FM897290	FM897273	KR856075	JF699308
*Indopolystoma elongatum* n. sp.	*Zhangixalus arboreus*	Rhacophoridae	Japan	AM157190	AM157213	KR856094	KR856170
*Indopolystoma parvum* n. sp.	*Z. omeimontis*	Rhacophoridae	China	AM157189	AM157212	KR856093	KR856169
*Indopolystoma viridi* n. sp.	*Z. viridis*	Rhacophoridae	Japan	AM157191	AM157214	KR856095	MN564839
Indopolystoma* sp.	*Z. smaragdinus*	Rhacophoridae	India	AM157193	AM157216	KR856085	MN564838
*Parapolystoma bulliense*	*Litoria gracilenta*	Hylidae	Australia	AM157186	AM157202	KR856079	KR856166
*Polystoma cuvieri*	*Physalaemus cuvieri*	Leptodactylidae	Paraguay	AM051068	AM157203	KR856080	AM913862
*Polystoma gallieni*	*Hyla meridionalis*	Hylidae	France	AM051070	AM157205	KR856084	JF699305
*Polystoma naevius*	*Smilisca baudinii*	Hylidae	Costa Rica	AM157187	AM157209	KR856089	AM913864
*Pseudodiplorchis americanus*	*Scaphiopus couchii*	Scaphiopodidae	USA	AM051079	AM157219	KR856097	KR856173
*Pseudopolystoma dendriticum*	*Onychodactylus japonicus*	Hynobiidae	Japan	FM992700	FM992707	KR856122	KR856180

* This undescribed polystome species, which was recovered from *R. maximus* of India, was tentatively considered as *P. indicum* in Verneau et al. [51], Badets et al. [2] and Héritier et al. [22]. Its host species, which was originally considered as *R. maximus*, should bear the nomen *Z. smaragdinus*.

### Sequence analyses

18S and 28S sequences were aligned according to the procedure described in Badets et al. [2] and Héritier et al. [22] who took into account the rRNA secondary structure (stems and loops) of both genes. Partial COI and 12S gene sequences were aligned independently using Clustal W under default parameters [44] implemented in MEGA7 software [30]. Because it was too difficult to assess homologous characters within a highly variable region in the 12S, that specific region was deleted prior to running phylogenetic analyses. Using ModelTest implemented in PAUP* version 4.0b9 [43], evolutionary models were estimated independently for the four partitions from the Akaike Information Criterion [38]. All partitions with their own evolutionary model (18S: nst = 6; rates = invgamma; ngammacat = 4; 28S: nst = 6; rates = invgamma; ngammacat = 4; COI: nst = 2; rates = invgamma; ngammacat = 4; 12S: nst = 6; rates = gamma; ngammacat = 4) were subsequently concatenated and a Bayesian analysis was conducted using MrBayes 3.04b [24], with four chains running for one million generations and sampled every 100 cycles. Convergence was assessed with the program Tracer v1.7.1 (http://beast.community/tracer) [39]. A consensus tree was then reconstructed after removing the first 1000 trees (10%) as the burn-in phase. Finally, COI and 28S genetic divergences (*p*-distances) as well as total differences were also computed for species delimitations following thresholds designed in Du Preez et al. [17]. When all positions containing missing data and/or gaps were eliminated, there were a total of 342 and 1300 positions in the final COI and 28S datasets, respectively.

## Results

### Phylogenetic relationships and genetic differentiation within polystomes

**Figure 1:**
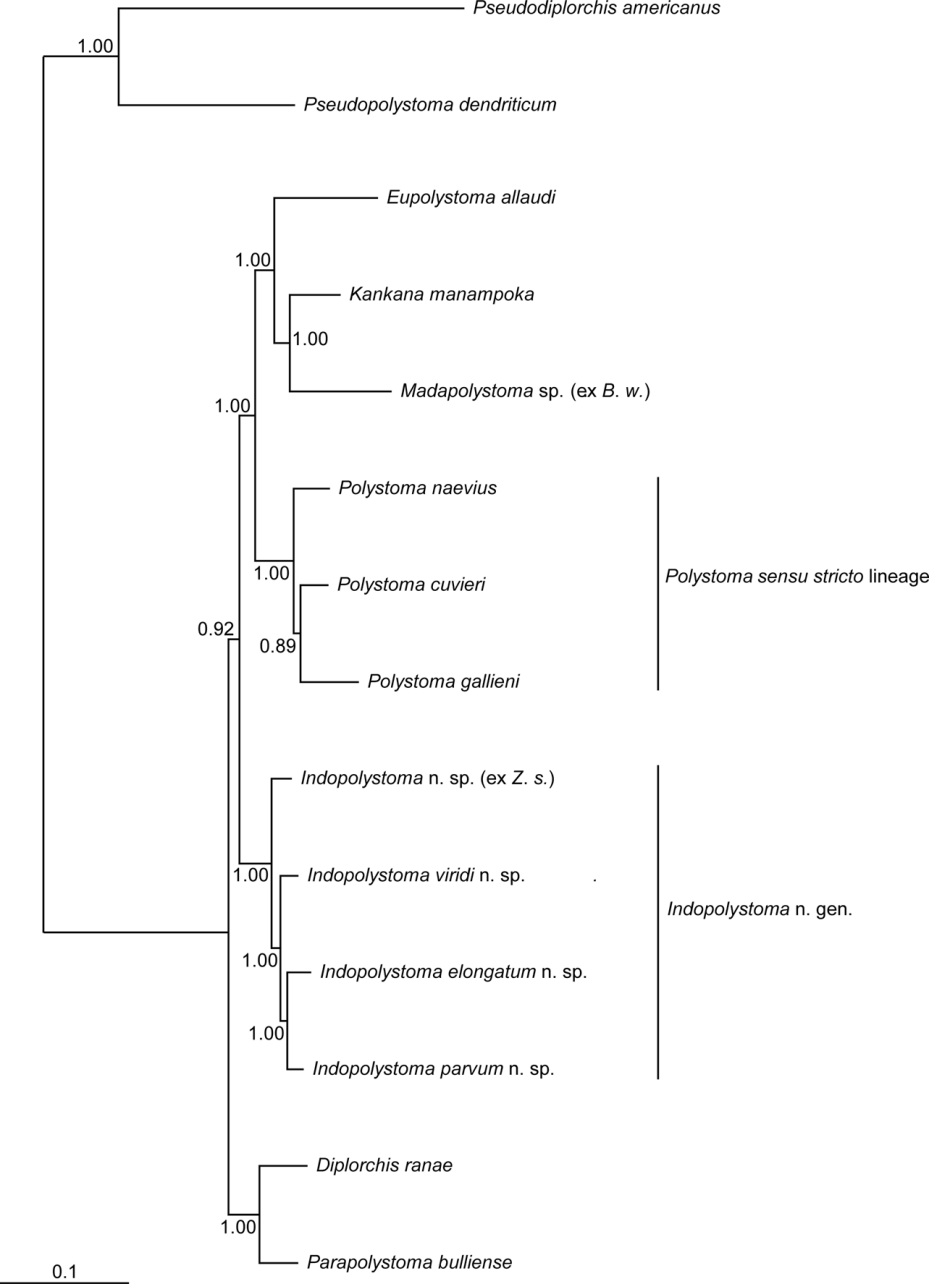
Bayesian tree for neobatrachian polystomes inferred from the analysis of four concatenated genes, namely 18S, 28S, COI and 12S. Numbers on nodes indicate Bayesian Posterior Probabilities. *Indopolystoma* spp. were regarded earlier as *Polystoma* spp. in Badets et al. [2] and Héritier et al. [22]. *B. w.* refers to *Blommersia wittei* and *Z. s.* to *Zhangixalus smaragdinus*. See also Table 1 for other host species. Scale bar represents 0.1 substitution/site.

The Bayesian tree (Fig. 1) shows phylogenetic relationships within polystomes of the Neobatrachia. As previously illustrated by Badets et al. [2], *Polystoma* appears paraphyletic, the *Polystoma sensu stricto* lineage being more closely related to a clade grouping *Eupolystoma*, *Kankana* Raharivololoniaina et al. 2011 and *Madapolystoma* Du Preez et al. 2010 than it is to the other *Polystoma* lineage called here for more convenience *Indopolystoma* n. gen. Regarding the genetic differentiation between that clade and the *Polystoma sensu stricto* lineage, which is about 18.2% in the COI (Table 2) and 3.7% in the 28S (Table 3), we can indeed consider it is a new genus according to its morphological characteristics (see below).

**Table 2:** Matrix of COI *p*-distances (lower left) and total differences (upper right) inferred from pairwise comparisons in MEGA7. *B. w.* refers to *B. wittei* and *Z. s.* to *Z. smaragdinus*. See also Table 1 for other host species.

	[1]	[2]	[3]	[4]	[5]	[6]	[7]	[8]	[9]	[10]	[11]	[12]	[13]	[14]
*Polystoma cuvieri* [1]	0	50	49	65	72	64	69	78	70	72	64	67	75	69
*Polystoma gallieni* [2]	0.146	0	55	64	66	61	64	78	67	73	67	71	80	69
*Polystoma naevius* [3]	0.143	0.161	0	51	55	54	64	70	69	67	60	66	77	70
*Indopolystoma* sp. (ex *Z. s.*) [4]	0.19	0.187	0.149	0	35	43	47	79	64	71	70	56	74	74
*Indopolystoma viridi* n. sp. [5]	0.211	0.193	0.161	0.102	0	36	50	75	67	75	69	64	70	71
*Indopolystoma parvum* n. sp. [6]	0.187	0.178	0.158	0.126	0.105	0	49	80	68	80	63	69	79	75
*Indopolystoma elongatum* n. sp. [7]	0.202	0.187	0.187	0.137	0.146	0.143	0	78	68	75	75	67	74	73
*Eupolystoma alluaudi* [8]	0.228	0.228	0.205	0.231	0.219	0.234	0.228	0	71	77	76	78	89	78
*Kankana manampoka* [9]	0.205	0.196	0.202	0.187	0.196	0.199	0.199	0.208	0	56	65	66	77	77
*Madapolystoma* sp. (ex *B. w.*) [10]	0.211	0.213	0.196	0.208	0.219	0.234	0.219	0.225	0.164	0	78	77	71	87
*Diplorchis ranae* [11]	0.187	0.196	0.175	0.205	0.202	0.184	0.219	0.222	0.19	0.228	0	65	72	76
*Parapolystoma bulliense* [12]	0.196	0.208	0.193	0.164	0.187	0.202	0.196	0.228	0.193	0.225	0.19	0	71	78
*Pseudodiplorchis americanus* [13]	0.219	0.234	0.225	0.216	0.205	0.231	0.216	0.26	0.225	0.208	0.211	0.208	0	82
*Pseudopolystoma dendriticum* [14]	0.202	0.202	0.205	0.216	0.208	0.219	0.213	0.228	0.225	0.254	0.222	0.228	0.24	0

**Table 3:** Matrix of 28S *p*-distances (lower left) and total differences (upper right) inferred from pairwise comparisons in MEGA7. *B. w.* refers to *B. wittei* and *Z. s.* to *Z. smaragdinus*. See also Table 1 for other host species.

	[1]	[2]	[3]	[4]	[5]	[6]	[7]	[8]	[9]	[10]	[11]	[12]	[13]	[14]
*Polystoma cuvieri* [1]	0	29	17	42	41	42	41	58	55	79	59	58	232	206
*Polystoma gallieni* [2]	0.022	0	38	57	54	55	54	79	73	94	69	69	231	206
*Polystoma naevius* [3]	0.013	0.029	0	49	48	49	48	64	60	80	69	65	233	205
*Indopolystoma* sp. (ex *Z. s.*) [4]	0.032	0.044	0.038	0	5	6	5	60	56	82	49	43	233	195
*Indopolystoma viridi* n. sp. [5]	0.032	0.042	0.037	0.004	0	3	2	59	57	81	50	44	221	196
*Indopolystoma parvum*. n. sp. [6]	0.032	0.042	0.038	0.005	0.002	0	3	60	58	82	51	45	223	196
*Indopolystoma elongatum* n. sp. [7]	0.032	0.042	0.037	0.004	0.002	0.002	0	59	57	81	50	44	223	196
*Eupolystoma alluaudi* [8]	0.045	0.061	0.049	0.046	0.045	0.046	0.045	0	50	79	75	70	241	214
*Kankana manampoka* [9]	0.042	0.056	0.046	0.043	0.044	0.045	0.044	0.038	0	66	69	59	239	211
*Madapolystoma* sp. (ex *B. w.*) [10]	0.061	0.072	0.062	0.063	0.062	0.063	0.062	0.061	0.051	0	89	89	231	205
*Diplorchis ranae* [11]	0.045	0.053	0.053	0.038	0.038	0.039	0.038	0.058	0.053	0.068	0	20	227	197
*Parapolystoma bulliense* [12]	0.045	0.053	0.05	0.033	0.034	0.035	0.034	0.054	0.045	0.068	0.015	0	228	193
*Pseudodiplorchis americanus* [13]	0.178	0.178	0.179	0.172	0.17	0.172	0.172	0.185	0.184	0.178	0.175	0.175	0	176
*Pseudopolystoma dendriticum* [14]	0.158	0.158	0.158	0.15	0.151	0.151	0.151	0.165	0.162	0.158	0.152	0.148	0.135	0

Taking into account that uncorrected *p*-distances estimates within Asian polystomes are well beyond 10% in the COI (Table 2) and 0.2% in the 28S (Table 3), we can assume that there are four separate species according to the 1.2% and 0.07% genetic divergences that were considered as the species-level threshold within polystomes of amphibians from COI and 28S sequences, respectively [17]. Furthermore, though several substitutions were found between Asian polystomes, at least one unique change (autapomorphy) was observed in each of the four undescribed species, regardless of the gene of interest, COI or 28S. These results reinforced our hypothesis of four distinct polystome species.

Finally, a Bayesian tree inferred from the analysis of a dataset comprising 14 full-length 18S sequences (Table 1), which was supplemented by unpublished partial 18S sequences obtained from *Polystoma carvirostris* Fan, Li & He, 2008 (EU734835), *P. zuoi* Shen, Wang & Fan, 2013 (KF850147) and two other undescribed *Polystoma* spp. infecting *Rana chaochiaoensis* Liu (U734834) and *Hyla annectans* (Jerdon) (EU979386) of China, showed that the last two species were more closely related to species of *Polystoma* than they were to species of *Indopolystoma* (results not shown).

### Morphological analyses with the description of a new genus and three new species within polystomes

#### *Indopolystoma* n. gen.

urn:lsid:zoobank.org:act:35517B78-23E5-4976-8D47-776F62A5D82F

##### Generic diagnosis

Body large and oblong. Intestical caeca bifurcate, diverticulated, confluent posteriorly with posterior diverticulum barely entering haptor. Intestinal anastomoses usually absent but at most a single anastomosis may be present. Vas deferens extends antero-medially, opens into seminal vesicle that opens into genital bulb, armed with 8–9 genital spines. Ovary comma shaped and prominent, sinistral, in anterior 20% of body. Oviduct arises from posterior region of ovary, connected by genito-intestinal canal to sinistral caecum, receives common vitelline duct, ascends giving rise to short tubular uterus that often holds a single egg but as many as 40. Vitellaria distributed throughout body proper except in region around ovary and reproductive ducts, extending marginally into haptor; left and right vitelline ducts join to form common vitelline reservoir near ovary, with duct to oviduct. Two prominent vaginae, antero-lateral to ovary; left and right vaginal ducts connected to respective vitelline ducts. Egg operculate, oval and lacking a filament. Haptor short relative to body size (haptor/total body length ratio < 0.15 for most species) with three pairs of suckers, one pair of hamuli and 16 marginal hooklets. Hamuli curved, unbranched in base (handle and guard not well separated) and with short recurved hook. Prominent big posteriormost marginal hooklet C1 (see numbering, [36]) with prominent broad blade and guard, in contrast with smaller hooklets C2–C8. *Indopolystoma* spp. are parasites of the bladder of rhacophorid frogs from Asia.

Etymology: The prefix indo refers to India, which was assumed to be the center of origin for this new genus [2].

Gender: neuter.

Type-species: *Indopolystoma viridi* n. sp.

Other species: *Indopolystoma elongatum* n. sp., *Indopolystoma parvum* n. sp., *Indopolystoma carvirostris* (Fan, Li & He, 2008) n. comb., *Indopolystoma hakgalense* (Crusz & Ching, 1975) n. comb., *Indopolystoma indicum* (Diengdoh & Tandon, 1991) n. comb., *Indopolystoma leucomystax* (Zhang & Long, 1987) n. comb., *Indopolystoma mutus* (Meng, Song & Ding, 2010) n. comb., *Indopolystoma pingbianensis* (Fan, Wang & Li, 2004) n. comb., *Indopolystoma rhacophori* (Yamaguti, 1936) n. comb., *Indopolystoma zuoi* (Shen, Wang & Fan, 2013) n. comb., and *Indopolystoma* sp.

##### Differential diagnosis

**Figure 2:**
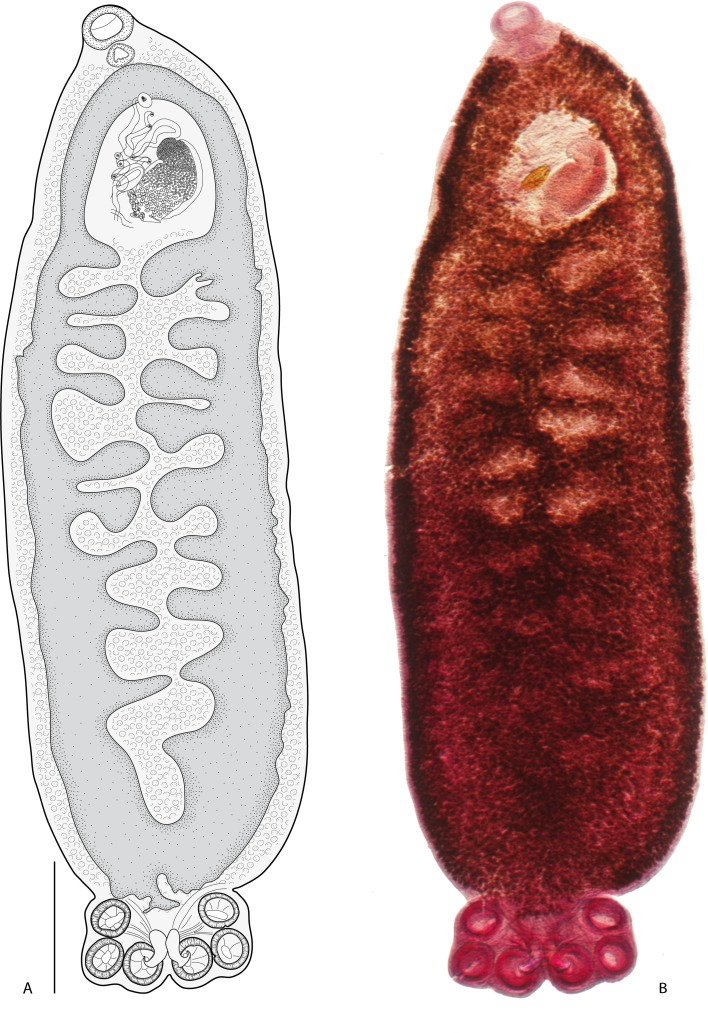
*Indopolystoma viridi* n. gen. n. sp. from *Zhangixalus viridis*. Dorsal view of holotype. (A) A drawing; (B) A photograph. Scale bar: 1 mm.

**Figure 3:**
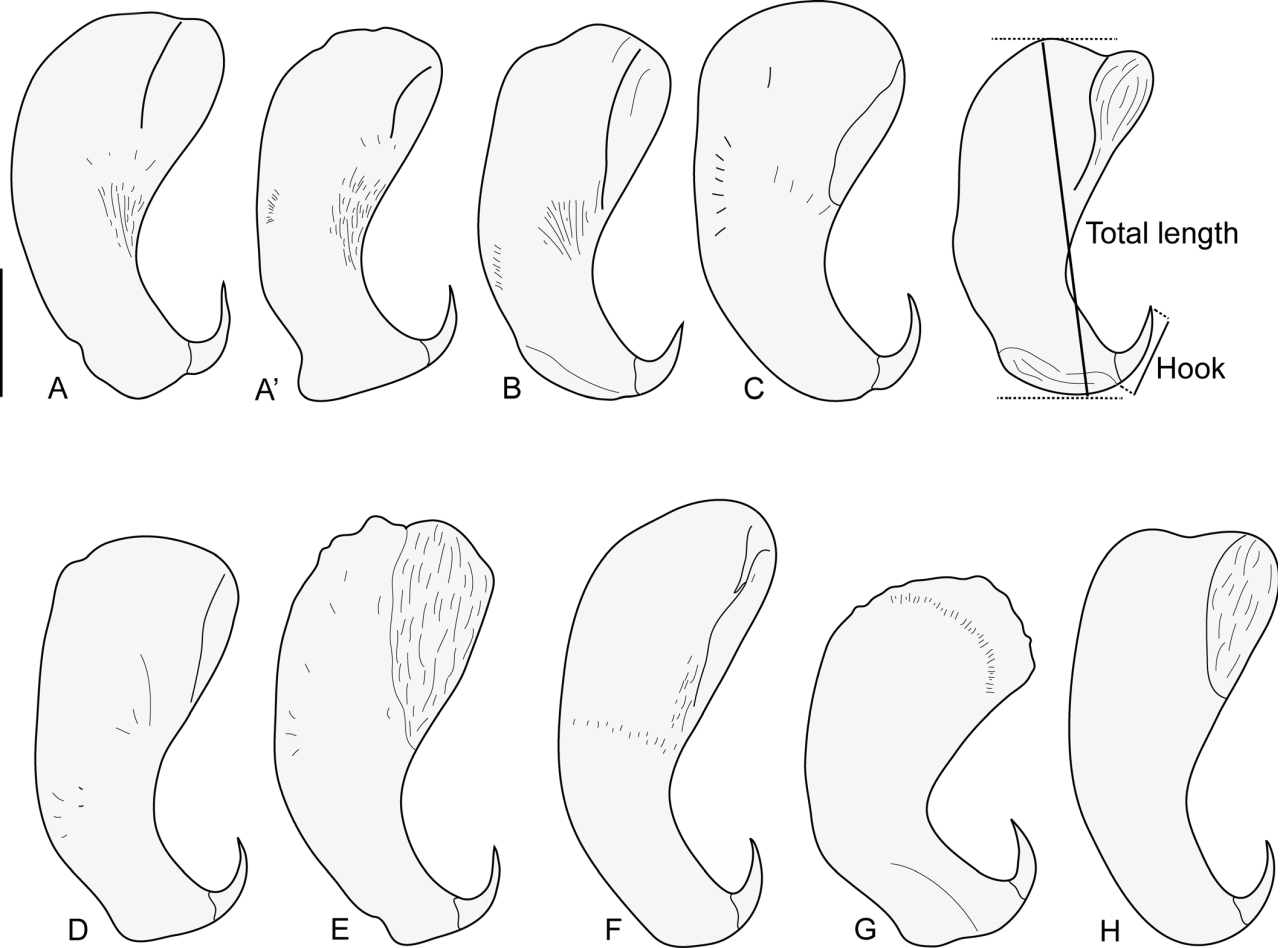
*Indopolystoma viridi* n. gen. n. sp. from *Zhangixalus viridis*. Hamuli from holotype and paratypes. (A–A’) Holotype; (B–H) Paratypes. Scale bar: 100 μm.

**Figure 4:**
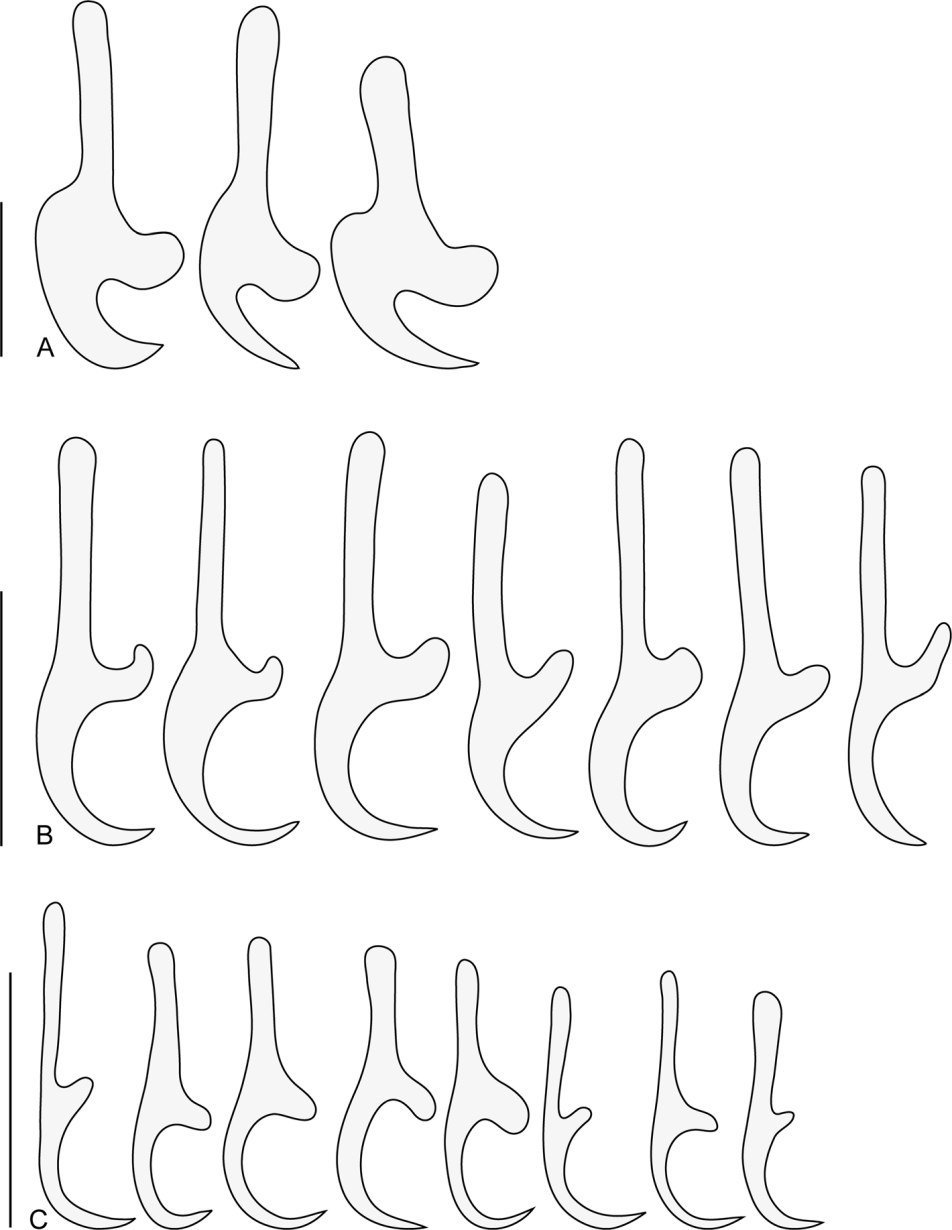
*Indopolystoma viridi* n. gen. n. sp. from *Zhangixalus viridis*. Marginal hooklets from paratypes. (A) Marginal hooklet C1; (B) Marginal hooklet C8; (C) Marginal hooklets C2–C7. Scale bar: 20 μm.

**Figure 5:**
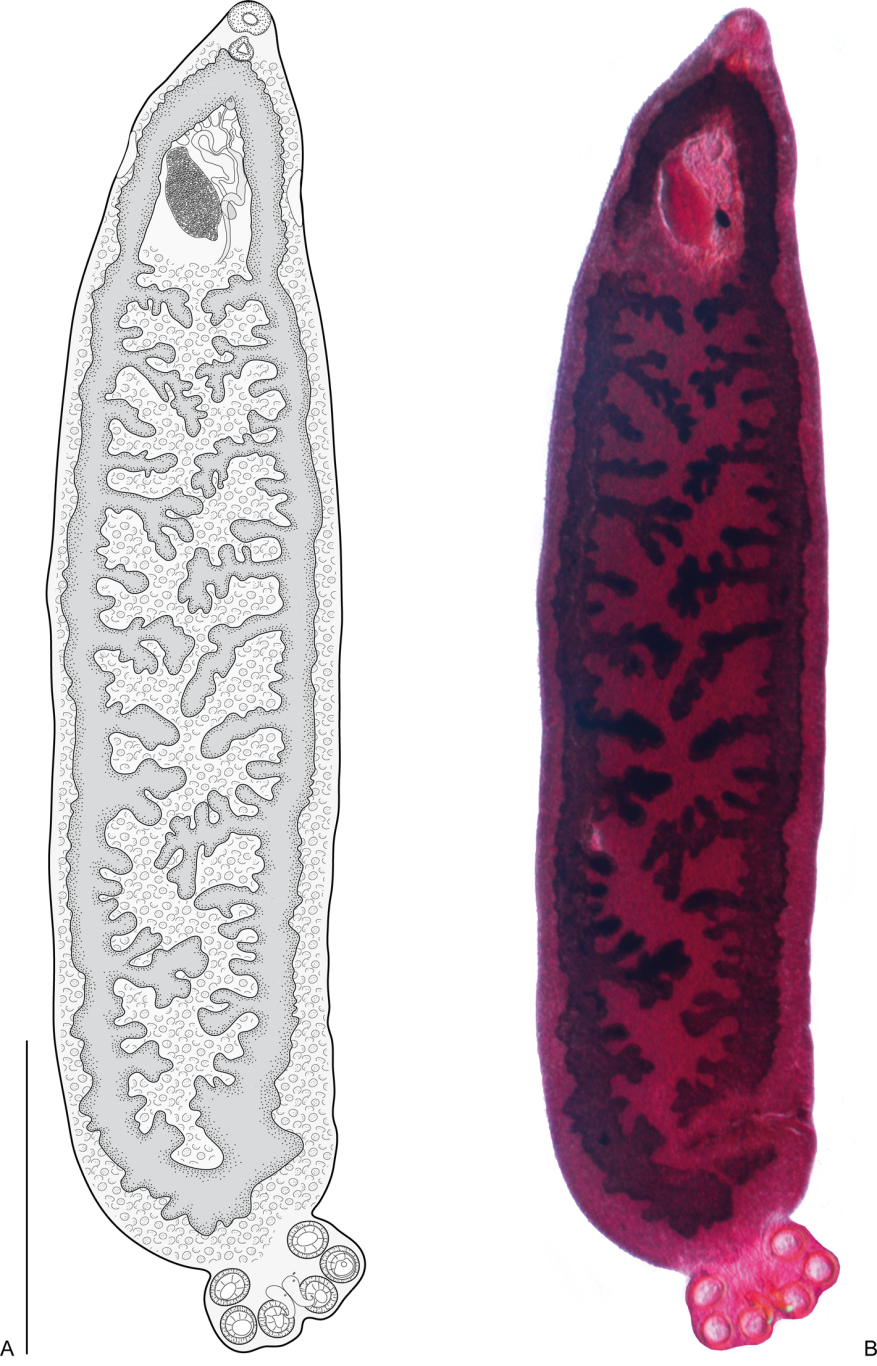
*Indopolystoma elongatum* n. gen. n. sp. from *Zhangixalus arboreus*. Ventral view of holotype. (A) A drawing; (B) A photograph. Scale bar: 1 mm.

**Figure 6:**
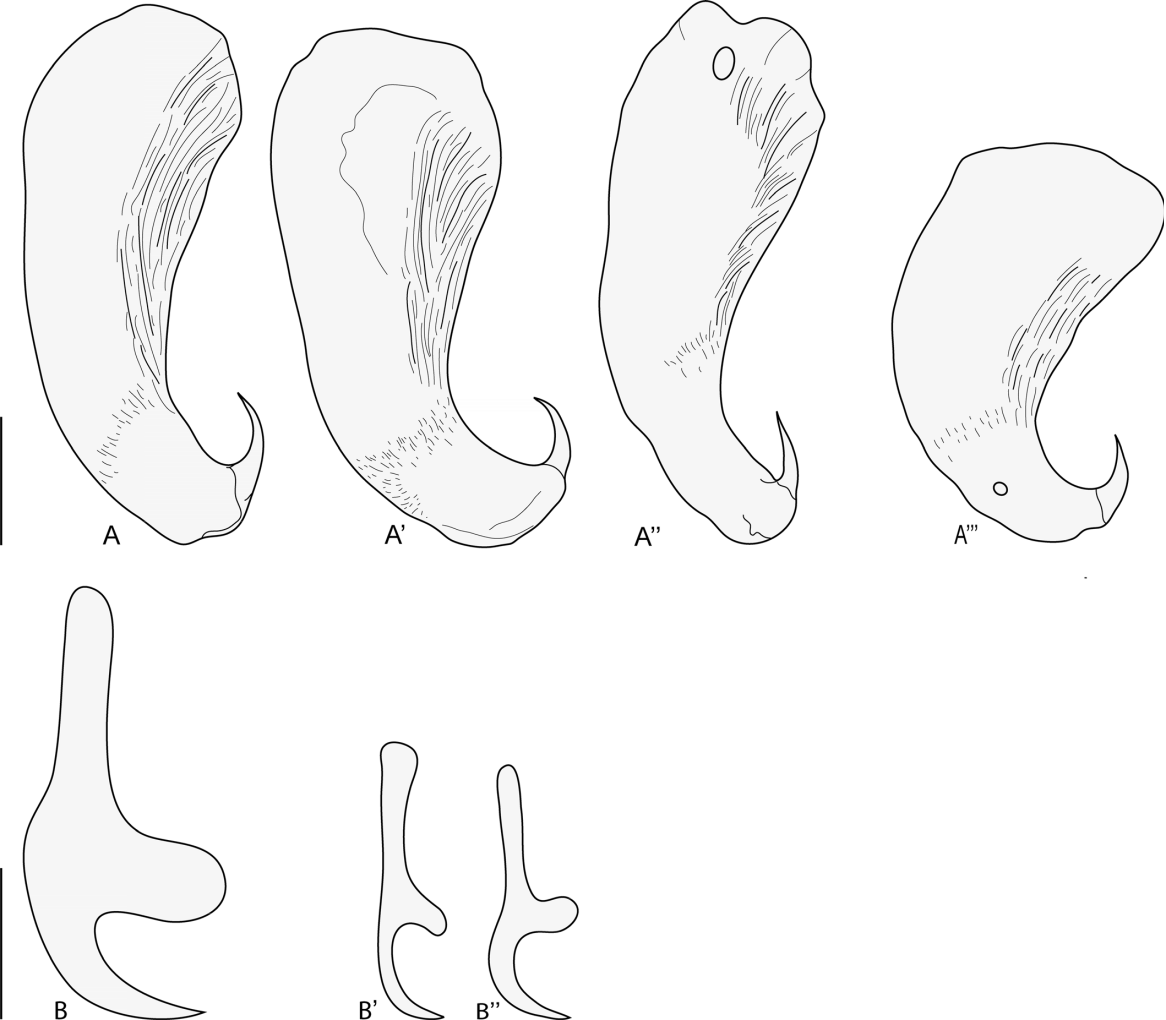
*Indopolystoma elongatum* n. gen. n. sp. from *Zhangixalus arboreus*. Hamuli and marginal hooklets from holotype and paratypes. (A–A’) Holotype; (A’’–A’’’) Paratypes; (B) Marginal hooklet C1; (B’) Marginal hooklet C3; (B’’) Marginal hooklet C8. Scale bar: 100 μm (A–A’’’), 20 μm (B–B’’).

**Figure 7:**
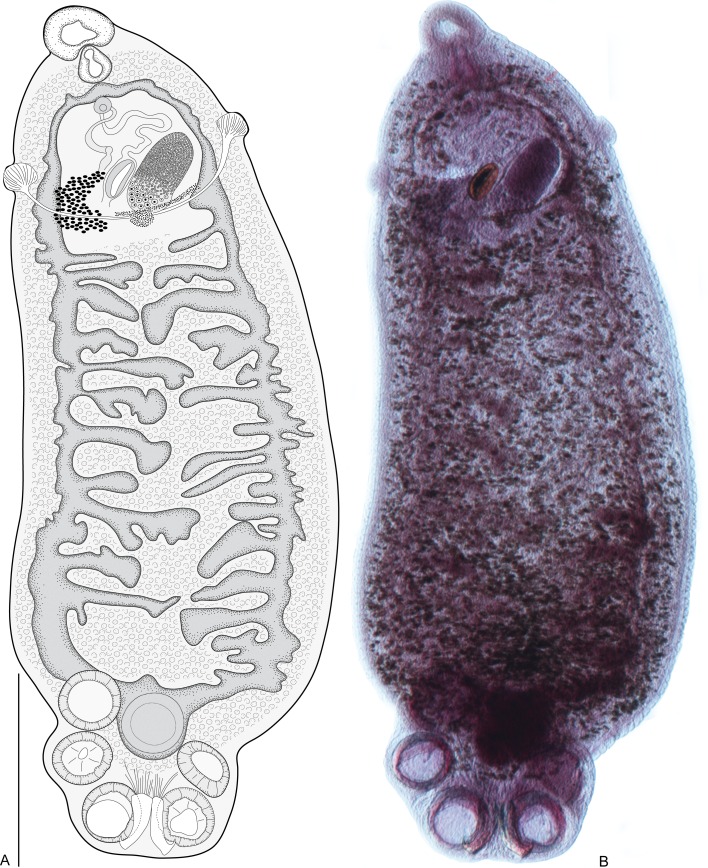
*Indopolystoma parvum* n. gen. n. sp. from *Zhangixalus omeimontis*. Dorsal view of holotype. (A) A drawing; (B) A photograph. Scale bar: 1 mm.

**Figure 8:**
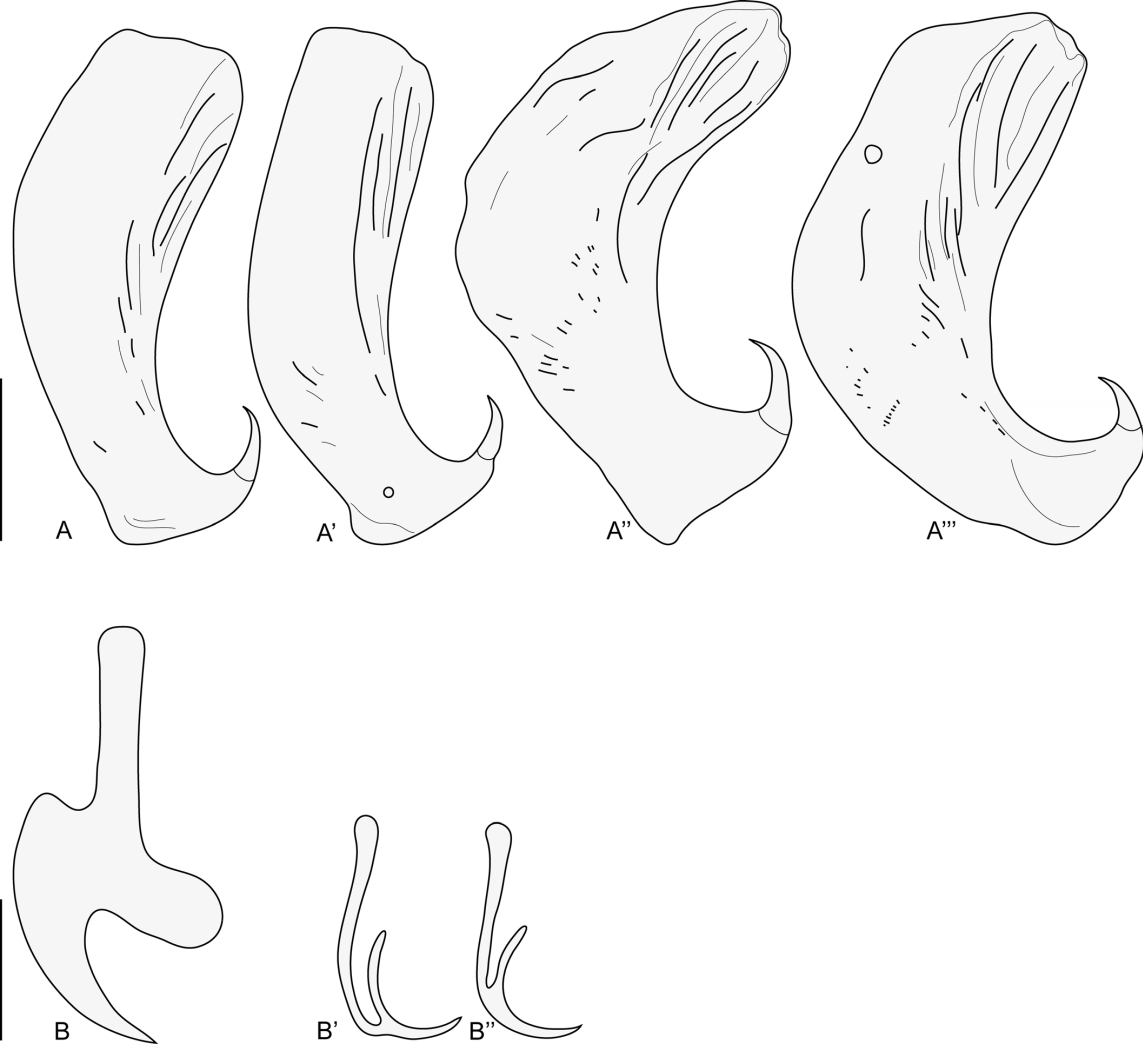
*Indopolystoma parvum* n. gen. n. sp. from *Zhangixalus omeimontis*. Hamuli and marginal hooklets from holotype and paratype. (A–A’) Holotype; (A’’–A’’’) Paratype; (B) Marginal hooklet C1; (B’) Marginal hooklet C2; (B’’) Marginal hooklet C6 or C7. Scale bar: 100 μm (A–A’’’), 20 μm (B–B’’).

Within Asia, *Indopolystoma* can be distinguished from other polystomatid genera infecting anurans by a combination of characteristics. Unlike *Diplorchis*, *Eupolystoma* and *Sundapolystoma* that all have an extensive uterus, it has a short uterus like *Polystoma* and *Neoriojatrema*. Unlike *Eupolystoma* and *Neoriojatrema* that lack hamuli, it has a single pair of hamuli like *Polystoma*, *Diplorchis* and *Sundapolystoma*. The haptor/total body length ratio is, for all species but one, less than 0.15 while it is usually far greater for all other anuran polystomes, namely *Polystoma* (0.19–0.27), *Diplorchis* (0.15–0.29), *Eupolystoma* (0.15–0.34), *Neoriojatrema* (0.34) and *Sundapolystoma* (0.28). Whereas *Eupolystoma*, *Neoriojatrema* and *Sundapolystoma* all have marginal hooklets of equal length, posteriormost marginal hooklet C1 in *Indopolystoma*, *Polystoma* and *Diplorchis* is bigger than the remainder. However, if the posteriormost marginal hooklet C1 is the same shape as hooklets C2–C8 in *Polystoma* and *Diplorchis*, it is far more developed with prominent broad blade and guard in *Indopolystoma* (Table 4, Figs. 2–8).

**Table 4:** Body measurements of *Indopolystoma* spp.

Parasite species	*Indopolystoma viridi* n. sp.	*Indopolystoma elongatum* n. sp.	*Indopolystoma parvum* n. sp.	*Indopolystoma carvirostris* n. comb.	*Indopolystoma hakgalense* n. comb.	*Indopolystoma indicum* n. comb.	*Indopolystoma leucomystax* n. comb.	*Indopolystoma mutus* n. comb.	*Indopolystoma pingbianensis* n. comb.	*Indopolystoma rhacophori* n. comb.	*Indopolystoma zuoi* n. comb.

Host species	*Zhangixalus viridis*	*Zhangixalus arboreus*	*Zhangixalus omeimontis*	*Kurixalus bisacculus* or *Kurixalus verrucosus*	*Taruga eques*	*Rhacophorus nigropalmatus*	*Polypedates leucomystax*	*Polypedates mutus*	*Zhangixalus dugritei*	*Zhangixalus arboreus*	*Feihyla palpebralis*
Site	Bladder	Bladder	Bladder	Bladder	Bladder	Bladder	Bladder	Bladder	Bladder	Bladder	Bladder
Locality	Japan	Japan	China	China	Sri Lanka	India	China	China	China	Japan	China
Number of mature specimens	15	3	2	10	12	8	5	2	6–7	–	3
Body length	8550 (5532–11,907)	14,791 (12,847–14,878)	4714‬ (4536–4891)	5070 (4030–7790)	‬(3870–9230)	12,556 (6732–16,614)	7568 (6665–8514)	7338 (6875–7800)	9428 (6000–12,000)	(4500–6000)	2716 (1624–3533)
Greatest width	2112 (1534–2859)	3182 (3170–3270)	1916‬ (1701–2130)	1130 (810–1400)	(890–1790)	4164 (2222–5641)	2732 (2542–3311)	3063 (2550–3575)	1990 (1640–2390)	‬(1000–1900)	1280 (1202–1904)
Width at vagina	1481 (1175–1800)	1967 (1902–2031)	1402 (1279–1525)	830	–	2057 (1197–2622)	–	–	1572	–	–
Haptor length	773 (526–1354)	916 (734–1098)	667‬ (584–749)	680 (500–750)	(635–794)	1298 (977–1710)	824 (753–989)	913 (875–950)	869 (424–1253)	(800–1100)	701 (632–924)
Haptor width	1184 (588–1592)	1661 (1512–1810)	1258‬ (1022–1494)	890 (640–1500)	‬(1016–1509)	2104 (1584–2466)	1693 (1355–2040)	1650 (1425–1875)	784 (415–1374)	(‬1100–1600)	1063 (831–1317)
False oral sucker width	394 (266–465)	551 (528–573)	395‬ (358–431)	–	(189–402)	466 (236–574)	392 (194–424)	388 (275–500)	–	–	–
Pharynx length	210 (124–268)	237 (227–247)	191‬ (187–194)	–	–	291 (164–395)	252 (219–286)	245 (210–280)	253 (218–328)	(188–240)	164
Pharynx width	209 (164–244)	259 (256–268)	190‬ (174–205)	–	(135–197)	249 (164–349)	216 (194–246)	235 (200–270)	229 (189–252)	‬(188–220)	151
Ovary length	775 (577–925)	1072 (1062–1108)	545‬ (483–606)	497	‬(492–874)	1049 (504–1368)	608 (451–683)	588 (550–625)	711.5 (453–927)	(420–630)	369 (329–400)
Ovary width	401 (286–536)	520 (514–563)	269‬ (231–306)	390	‬(207–349)	499 (288–630)	259 (191–294)	400 (375–425)	–	‬(210–340)	–
Length of eggs	241 (191–268)	240 (224–256)	221‬ (219–222)	–	–	205 (190–214)	–	–	307	(238–300)	–
Width of eggs	125 (74–165)	118 (106–130)	103 (93–113)	–	–	132 (125–140)	–	–	165	‬(140–163)	–
Number of intra-uterine eggs	1	1	1	0	–	up to 40	0	0	1–8	Usually 1 but sometimes more than 10	0
Genital bulb width	105 (67–124)	139 (129–211)	83 (75–90)	–	–	–	(69–76)	–	–	–	–
Number of genital spines	8–9	8	8	8	–	8	8	9	–	8	8
Length of genital spine	40 (27–49)	41 (38–44)	17 (16–18)	–	–	39 (38–40)	–	39 (38–40)	–	42	–
Haptoral sucker width	333 (242–423)	420‬ (380–459)	335‬ (281–389)	259 (212–339)	‬(250–320)	353 (264–465)	289 (207–356)	310 (270–350)	434 (349–491)	(320–400)	251 (209–311)
Hamulus handle length	313 (276–373)	407 (303–419)	326‬ (311–340)	285 (257–326)	(380–440)	320 (178–414)	332 (260–410)	340 (330–350)	408 (332–441)	‬(350–420)	240 (185–307)
Hamulus guard length	–	–	–	249 (208–306)	–	–	–	290 (280–300)	382 (340–461)	–	205 (173–265)
Hamulus hook length	66 (48–74)	78.5‬ (72–85)	52‬ (39–64)	–	–	–	–	40 (38–43)	–	–	–
Marginal hooklet length	–	–	–	(16–18) only on suckers	(20.5–36.9) C1–C2	–	–	–	–	(24–42)	–
Marginal hooklet C1 length	40 (31–44)	36	32	–	–	–	–	–	–	(38–42)	–
Marginal hooklet C2–C8 length	21.5 (16–31)	23 (18–32)	(19–20)	–	–	–	–	–	–	(24–27)	–
Haptoral length /Body length	0.09 (0.05–0.17)	0.06 (0.05–0.07)	0.14 (0.12–0.165)	0.13	0.11	0.10	0.11	0.12	0.09	0.18	0.26
Number of anastomoses	0	>1	1	>1	>1	1–2	>1	>1	0–1	>1	3–4

Note. To the exception of the newly described species, body measurements for all other species were extracted or estimated from Crusz and Ching [12], Diengdoh and Tandon [13], Fan et al. [19, 20], Meng et al. [33], Shen et al. [40], Yamaguti [53] and Zhang and Long [54].

“–” means data missing.

#### *Indopolystoma viridi* n. gen. n. sp. (Figs. 2, 3 and 4; Table 4)

urn:lsid:zoobank.org:act:E314A3B7-A5CE-48BA-9A21-B100556A34B9

Synonym: *Polystoma* sp. of Verneau et al. [51], Badets et al. [2] and Héritier et al. [22].

Type-host: *Rhacophorus viridis* (Hallowell). Now *Zhangixalus viridis* (Hallowell) [21, 27].

Site: Bladder.

Type-locality: Tokunoshima Island, Kagoshima prefecture, Japan.

Collector: Professor Hideo Hasegawa, Department of Biology, Oita Medical University, Hasama, Oita 879-5593, Japan.

Type-specimens: Holotype (MNHN HEL1173) and 10 paratypes (MNHN HEL1174–HEL1183) deposited in the Parasite Collection, Muséum national d’Histoire naturelle, Paris, France and four paratypes (NMB P512–P515) in the Parasitic Worm Collection, National Museum, Aliwal Street, Bloemfontein, South Africa.

Etymology: The name *viridi* refers to the species name of its host.

##### Description

Description based on 15 unflattened specimens stained in carmine and mounted in Canada balsam. Body elongate, total length 8550 (5532–11,907), including haptor; greatest width 2112 (1534–2859); width at vagina 1481 (1175–1800). Tegument smooth. Haptor sub-rectangular, 773 (526–1354) long, 1184 (588–1592) wide. Haptor/total body length ratio 0.09 (0.05–0.17). Suckers 333 (242–423) in diameter. Hamuli 313 (276–373) long; with hook 66 (48–74) long (Fig. 3A–H). Marginal hooklet C1 40 (31–44) long; C2–C8 21.5 (16–31) long (Fig. 4A–C). Mouth ventral, sub-terminal and surrounded by false oral sucker; false oral sucker 394 (266–465) wide. Pharynx pyriform, 210 (124–268) long, 209 (164–244) wide. Oesophagus not visible. Lateral intestinal caeca with medial diverticula branched lacking prehaptoral and haptoral anastomoses. Testis not visible hidden by digestive tract and vitellaria. Seminal vesicle prominent and packed with sperm. Genital bulb slightly sclerotized, medio-ventral, 105 (67–124) in diameter, with eight to nine sclerotized genital spines; genital spines 40 (27–49) long. Ovary prominent, sinistral and packed with oocytes; ovary 775 (577–925) long, 401 (286–536) wide. Ootype well developed. Genito-intestinal canal present on the same side of body as ovary, joining intestinal caecum posterior to ovary. Uterus confined to area anterior to ovary holding one egg; egg 241 (191–268) long, 125 (74–165) wide. No intrauterine development of eggs observed (Fig. 2).

##### Differential diagnosis

*Indopolystoma viridi* is similar to *I. elongatum* and *I. parvum* in terms of body shape, haptor/total body length ratio and shape of haptoral sclerites. However, it differs from the same two species by the general morphology of intestinal caeca and its body size (8550 μm vs. 14,791 μm for *I. elongatum* and 4714 μm for *I. parvum*). It differs from all other species of *Indopolystoma* in having intestinal diverticula without anastomoses.

#### *Indopolystoma elongatum* n. gen. n. sp. (Figs. 5 and 6; Table 4)

urn:lsid:zoobank.org:act:EDFE29A4-9B26-4A25-A92A-BB3CF106D4EC

Synonym: *Polystoma* sp. of Badets et al. [2] and Héritier et al. [22].

Type-host: *Rhacophorus arboreus* (Okada & Kawano). Now *Zhangixalus arboreus* (Okada and Kawano) [21, 27].

Site: Bladder.

Type-locality: Upstream of Kunigami-gun, city of Nago, Okinawa prefecture, Japan.

Other localities: Sado Island, Niigata prefecture, Japan.

Collector: Professor Hideo Hasegawa, Department of Biology, Oita Medical University, Hasama, Oita 879-5593, Japan.

Type-specimens: Holotype (MNHN HEL1184) and 2 paratypes (MNHN HEL1185–HEL1186) deposited in the Parasite Collection, National Museum of Natural History, Paris, France.

Etymology: The species name *elongatum* refers to its general body shape, which is elongate.

##### Description

Description based on three flattened specimens stained in carmine and mounted in Canada balsam. Body elongate, tapered anteriorly, total length 14,791 (12,847–14,878), including haptor; greatest width 3182 (3170–3270); width at vagina 1967 (1902–2031). Tegument smooth. Haptor sub-rectangular, 916 (734–1098) long, 1661 (1512–1810) wide. Haptor/total body length ratio 0.06 (0.05–0.07). Suckers 420 (380–459) in diameter. Hamuli 407 (303–419) long; with hook 78.5 (72–85) long (Fig. 6A–A’’’). Marginal hooklet C1 36 long; C2–C8 23 (18–32) long (Fig. 6B–B’’). Mouth ventral, sub-terminal and surrounded by false oral sucker; false oral sucker 551 (528–573) wide. Pharynx pyriform, 237 (227–247) long, 259 (256–268) wide. Oesophagus not visible. Intestine bifurcate with medial diverticula highly branched giving rise to prehaptoral anastomoses (up to two). Testis not visible hidden by the digestive tract and vitellaria. Seminal vesicle prominent and packed with sperm. Genital bulb slightly sclerotized, medio-ventral, 139 (129–211) in diameter, with eight sclerotized genital spines; genital spines 41 (38–44) long. Ovary prominent, submedian and packed with oocytes; ovary 1072 (1062–1108) long, 520 (514–563) wide. Ootype well developed. Genito-intestinal canal present on same side of body as ovary, joining intestinal caecum posterior to ovary. Uterus confined to area anterior to ovary holding one egg; egg 240 (224–256) long, 118 (106–130) wide. No intrauterine development of eggs observed (Fig. 5).

##### Differential diagnosis

*Indopolystoma elongatum* is well characterized by its body size and shape. This species is much bigger and more elongated (body length 14,791 μm) than any other species of *Indopolystoma*, though there is an overlap of size values with *I. indicum*. *Indopolystoma elongatum* can be easily distinguished from the later by the number of intrauterine eggs. None of the specimens of *I. elongatum* have more than a single egg in utero while *I. indicum* has as many as 40.

Remarks: *Zhangixalus arboreus* hosts two polystomes, namely *I. elongatum* and *I. rhacophori* (see below), which is uncommon within anuran polystomes. However, *Z. arboreus* and *Z. schlegelii* occur sympatrically in Japan [1]. The possibility of a misidentification can thus not be excluded especially since molecular evidence on host identity is currently not available. We consider for now that both *I. elongatum* and *I. rhacophori* are separate species primarily on the basis of body length and haptor/total body length ratio (0.06 for *I. elongatum* vs. 0.18 for *I. rhacophori*).

#### *Indopolystoma parvum* n. gen. n. sp. (Figs. 7 and 8; Table 4)

urn:lsid:zoobank.org:act:6C7F74C6-BFEE-4277-903B-2A531FD09C63

Synonym: *Polystoma* sp. of Verneau et al. [51], Badets et al. [2] and Héritier et al. [22].

Type-host: *Rhacophorus omeimontis* (Stejneger). Now *Zhangixalus omeimontis* (Stejneger) [21, 27].

Site: Bladder.

Type-locality: Wawu Shan, Hongya Xian, Sichuan province, China.

Collector: Professor Anne Marie Ohler, Muséum National d’Histoire naturelle (MNHN), Paris, France.

Type-specimens: Holotype (MNHN HEL1187) and paratype (MNHN HEL1188) deposited in the Parasite Collection, National Museum of Natural History, Paris, France.

Etymology: The species name *parvum* refers to its small body size.

##### Description

Description based on two unflattened specimens stained in carmine and mounted in Canada balsam. Body elongate, total length 4714 (4536–4891), including haptor; greatest width 1916 (1701–2130); width at vagina 1402 (1279–1525). Haptor circular in outline, 667 (584–749) long, 1258 (1022–1494) wide. Haptor/total body length ratio 0.14 (0.12–0.165). Suckers 335 (281–389) in diameter. Hamuli 326 (311–340) long; with hook 52 (39–64) long (Fig. 8A–A’’’). Marginal hooklet C1 32 long; C2–C8 (19–20) long (Fig. 8B–B’’). Mouth ventral, sub-terminal and surrounded by false oral sucker; false oral sucker 395 (358–431) wide. Pharynx pyriform, 191 (187–194) long, 190 (174–205) wide. Oesophagus not visible. Intestine bifurcate with medial diverticula highly branched lacking prehaptoral anastomoses. Caeca confluent posteriorly, extending into haptor forming haptoral anastomosis. Testis lying on body midline anterior to ovary. Seminal vesicle prominent and packed with sperm. Genital bulb slightly sclerotized, medio-ventral, 83 (75–90) in diameter, with eight sclerotized genital spines; genital spines 17 (16–18) long. Ovary prominent, sinistral and packed with oocytes; ovary 545 (483–606) long, 269 (231–306) wide. Ootype well developed. Genito-intestinal canal present on same side of body as ovary, joining intestinal caecum posterior to ovary. Uterus confined to dextral and anterior to ovary holding one egg; egg 221 (219–222) long, 103 (93–113) wide. No intrauterine development of eggs observed (Fig. 7).

##### Differential diagnosis

*Indopolystoma parvum* can be easily distinguished from *I. viridi* and *I. elongatum* by its body size, haptor shape and general morphology of intestine. This species is much smaller than *I. elongatum* (4714 μm vs. 14,791 μm) while it is only half the size of *I. viridi* (4714 μm vs. 8550 μm). It shows haptor sub-spherical (vs. sub-rectangular) and intestinal caeca with haptoral anastomosis. It differs from all other congeners, apart from *I. pingbianensis*, in lacking medial anastomoses. *Indopolystoma parvum* is smaller than *I. pingbianensis* (4714 μm vs. 9428 μm).

#### *Indopolystoma carvirostris* (Fan, Li & He, 2008) n. comb. (Table 4)

Synonym: *Polystoma carvirostris* Fan, Li & He, 2008 [19].

Type-host: *Polypedates cavirostris* Günther. Now *Kurixalus bisacculus* (Taylor) (Previously *Rhacophorus bisacculus* Taylor) or *Kurixalus verrucosus* (Boulenger) (Previously *Rhacophorus verrucosus* Boulenger) [21, 26].

Site: Bladder.

Type-locality: Pingbian county (22°56′ N, 103°42′ E), Yunnan province, China.

Remarks: Although the authors of the original description did not draw the marginal hooklets [20], the general morphology of this species, including haptor/total body length ratio (0.13) and hamuli shape (unbranched), is consistent with the diagnosis of *Indopolystoma*. Furthermore, a phylogeny based on partial 18S sequences only (unpublished results) showed that this species fell within the clade of *Indopolystoma* species.

*Indopolystoma carvirostris* was originally recorded in China from *P. cavirostris*. However, *P. cavirostris* only occurs in Sri Lanka [21]. According to Inger et al. [26], Chinese records of *P. cavirostris* likely apply to *R. bisacculus* or *R. verrucosus*.

#### *Indopolystoma hakgalense* (Crusz & Ching, 1975) n. comb. (Table 4)

Synonym: *Polystoma hakgalense* Crusz & Ching, 1975 [12].

Type-host: *Rhacophorus cruciger eques* Kirtisinghe. Now *Taruga eques* (Günther) [21].

Site: Bladder.

Type-locality: Hakgala Strict Natural Reserve, Sri Lanka.

Remarks: Although the authors of the original description did not draw the marginal hooklets [12], the general morphology of this species, including haptor/total body length ratio (0.11) and hamuli shape (unbranched), is consistent with the diagnosis of *Indopolystoma*.

#### *Indopolystoma indicum* (Diengdoh & Tandon, 1991) n. comb. (Table 4)

Synonym: *Polystoma indicum* Diengdoh & Tandon, 1991 [13].

Type-host: *Rhacophorus nigropalmatus* Boulenger [21].

Site: Bladder

Type-locality: Cherrapunji (25°18′ N: 91°46′ E), East Khasi Hills District, Meghalaya state, India.

Remarks: Although the authors of the original description did not draw the marginal hooklets [13], the general morphology of this species, including haptor/total body length ratio (0.10) and hamuli shape (unbranched), is consistent with the diagnosis of *Indopolystoma*.

#### *Indopolystoma leucomystax* (Zhang & Long, 1987) n. comb. (Table 4)

Synonym: *Polystoma leucomystax* Zhang & Long, 1987 [54].

Type-host: *Polypedates leucomystax* (Gravenhorst) [21].

Site: Bladder.

Type-locality: Hangzhou, Zhejiang province, China.

Remarks: Although the authors of the original description did not draw the marginal hooklets [54], the general morphology of this species, including haptor/total body length ratio (0.11) and hamuli shape (unbranched), is consistent with the diagnosis of *Indopolystoma*.

#### *Indopolystoma mutus* (Meng, Song & Ding, 2010) n. comb. (Table 4)

Synonym: *Polystoma mutus* Meng, Song & Ding, 2010 [33].

Type-host: *Rhacophorus mutus* Smith. Now *Polypedates mutus* (Smith) [21].

Site: Bladder.

Type-locality: Jianfengling, Hainan Island, China.

Remarks: Although the authors of the original description did not draw the marginal hooklets [33], the general morphology of this species, including haptor/total body length ratio (0.12) and hamuli shape (unbranched), is consistent with the diagnosis of *Indopolystoma*.

#### *Indopolystoma pingbianensis* (Fan, Wang & Li, 2004) n. comb. (Table 4)

Synonym: *Polystoma pingbianensis* Fan, Wang & Li, 2004 [20].

Type-host: *Polypedates dugritei* David. Now *Zhangixalus dugritei* (David) [21, 27].

Site: Bladder.

Type-locality: Yunnan province, China.

Remarks: Although the authors of the original description did not draw the marginal hooklets [20], the general morphology of this species, including haptor/total body length ratio (0.09) and hamuli shape (unbranched), is consistent with the diagnosis of *Indopolystoma*.

#### *Indopolystoma rhacophori* (Yamaguti, 1936) n. comb. (Table 4)

Synonym: *Polystoma rhacophori* Yamaguti, 1936 [53].

Type-host: *Rhacophorus schlegelii* var*. arborea* Okada. Now *Zhangixalus arboreus* (Okada and Kawano) [21].

Site: Bladder.

Type-locality: Kurama, near Kyoto, Japan.

Remarks: Although the author of the original description did not draw the marginal hooklets [53], the general morphology of this species, including hamuli shape (unbranched), is consistent with the diagnosis of *Indopolystoma*. The haptor/total body length ratio of about 0.18 is bigger than that of any other *Indopolystoma* spp. with the exception of *I. zuoi* (Shen, Wang & Fan, 2013) n. comb. As discussed earlier for *I. elongatum*, which infests the same host, we consider for now that both *I. elongatum* and *I. rhacophori* are two separate species primarily on the basis of body length and haptor/ total body length ratio.

#### *Indopolystoma zuoi* (Shen, Wang & Fan, 2013) n. comb. (Table 4)

Synonym: *Polystoma zuoi* Shen, Wang & Fan, 2013 [40].

Type-host: *Philautus palpebralis* Smith. Now *Feihyla palpebralis* (Smith) [21].

Site: Bladder.

Type-locality: Pingbian county, Yunnan province (22°57.295′ N, 103°41.959′ E).

Remarks: Although the authors of the original description did not draw the marginal hooklets [40], the general morphology of this species, including hamuli shape (unbranched), is consistent with the diagnosis of *Indopolystoma*. The haptor/total body length ratio of about 0.26 is so much bigger than that of any other *Indopolystoma* spp. Nevertheless, a phylogeny based on partial 18S sequences only (unpublished results) showed that this species fell within the clade of *Indopolystoma* species.

#### *Indopolystoma* n. gen. sp.

Synonym: *Polystoma indicum* of Verneau et al. [51], Badets et al. [2] and Héritier et al. [22].

Type-host: *Rhacophorus maximus* Günther. Now *Zhangixalus smaragdinus* (Blyth) [21, 27].

Site: Bladder.

Type-locality: India.

Remarks: *Indopolystoma* sp. was tentatively assigned to *P. indicum* from *Z. smaragdinus* by Verneau et al. [51], Badets et al. [2] and Héritier et al. [22]. However, because we did not have any information on morphological characteristics of this species, which is nested in a clade with *I. viridi*, *I. elongatum* and *I. parvum* (Fig. 1; see also [2, 22, 51]), we must for now consider it as an undescribed species of *Indopolystoma*.

## Discussion

In this paper, one genus and three new species are being described, and eight previously described species of *Polystoma* as well as an undescribed species from *Z. smaragdinus* are being transferred to the new genus. Whereas species of *Polystoma* in Asia infect mostly ranids and hylids, species of *Indopolystoma* are only reported from species assigned to rhacophorid genera, such as *Feihyla, Kurixalus*, *Polypedates*, *Rhacophorus*, *Taruga* and *Zhangixalus*. These results clearly illustrate that rhacophorids are frequent hosts for *Indopolystoma* in Asia in which polystome speciation and diversification would have occurred during the long isolation of India from Madagascar and Africa. The Rhacophoridae is currently represented by 422 valid species arranged in 20 genera [1, 21]. As such, they account for roughly 6% of the living anurans of the world. These neobatrachians occur almost exclusively in India as well as in southeast Asia, with only one genus, *Chiromantis*, having species known from Africa [1, 21]. Therefore, we may expect a greater diversity of polystomes within *Indopolystoma* which should be restricted to Asia, where rhacophorids have undergone spectacular radiation “out of India” [32].

The interspecific morphological variation of polystomes is generally limited [45]. Herein, the haptor along with sclerotized structures (or sclerites) makes *Indopolystoma* a unique genus amongst all polystome genera infecting anuran hosts. Despite their morphological plasticity, the haptoral sclerites which are the “hallmark of monogeneans” [9] remain a significant character for morphological identification. Within amphibian polystomes, the haptoral sclerites are typically represented by 16 marginal hooklets and one pair of hamuli, although a few exceptions are known [14, 25, 31]. These characters have been largely investigated because of their usefulness in polystome delimitation [8, 10, 11, 14, 16, 25, 35, 36, 46–48]. The species of *Indopolystoma* are characterized by a posteriormost marginal hooklet C1, with prominent broad blade and guard, much larger than those of hooklets C2–C8, unlike that of *Polystoma* and *Diplorchis* spp. where the hooklets are all morphologically similar, although posteriormost marginal hooklet C1 is also larger than hooklets C2–C8. On the other hand, all marginal hooklets are equal in length and similar in shape within species of *Eupolystoma*, *Neoriojatrema* and *Sundapolystoma*. Whereas the presence of hamuli within *Indopolystoma* allows the differentiation of that genus from *Eupolystoma* and *Neoriojatrema* in which hamuli are lacking, their particular structure with a handle not separated from the guard, i.e. they lack a deep notch in base, is not unique as it is similar with some species of *Polystoma*. Finally, the haptor/total body length ratio is also of value for delimitating *Indopolystoma*. For all species of *Indopolystoma*, with the exception of *I. rhacophori* and *I. zuoi*, this value is less than 0.15, while it is greater for most other anuran polystomes. *Chiromantis rufescens* (Günther) is currently the only rhacophorid frog in Africa known to host a polystome, namely *Polystoma chiromantis* Dupouy & Knoepffler, 1978. Although marginal hooklets were not described in the original description [18], this parasite shares the elongated body and small haptor of *Indopolystoma*. According to Imasuen (unpublished thesis), marginal hooklet C1 of *P. chiromantis* has the typical shape as seen in *Polystoma* species. Therefore, in the absence of molecular evidence, we herein consider this species as belonging to *Polystoma*, which could have arisen from host-switching in Africa.

In conclusion, even though three main characters, i.e. the shape of the posteriormost marginal hooklet C1, the haptor/total body length ratio, and host species belonging to Rhacophoridae, constitute key characters for the morphological delimitation of *Indopolystoma*, it is important that genotyping of several polystome worms be conducted prior to the description process, as recommended by Héritier et al. [23].
